# Pediatric Aphorisms

**Published:** 1884-08-20

**Authors:** 


### PEDIATRIC APHORISMS.

The following aphorisms of Professor Letanundi are quoted in Le Dictanum of May 10th, 1884:

1. Children are like the mob: they always complain with reason, although they cannot give the reason why they complain.

2. Always look at the lips of a pale and sickly child; if they are of a deep red color, beware of prescribing tonics internally. At the outset you will congratulate yourself, but in the long run will repent of having employed them.

3. As a general rule, a sad child has an encephalic lesion; a furious child, an abdominal one; a soporific child has both, though indistinctly defined.

4. An attendance on children produces in the mind of an observant physician the conviction that the half, at least, of adult transgressions are so through morbid abdominal influences.

5. A sunny living-room, a clean skin, and an ounce of castor oil in the cupboard, these are three great points of infantile hygiene.

6. To dispute the clinical value of tracheotomy in croup is a waste of time to no good purpose. Croup or no croup, if there be a positive obstruction to respiration in the larynx, it is but according to reason to open a way for sub-laryngeal respiration. In the days of more knowledge and less nonsense, tracheotomy will be ranked among the minor surgical operations.

7. Dentition is a true multiple pregnancy, in which the uterus and its fœtuses become petrified in proportion as they grow. It is not the direct or eruptive pressure, but the lateral pressure of all together, that is most dangerous. It is from this that so many cerebral symptoms appear which can in no way be relieved by incisions of the gums. The only recourse against the dangers of transverse pressure is to give the child more nourishment, in the hope that as the general condition is bettered the local condition will also be improved.

8. If the incisors of the first dentition are serrated, it is bad; but if those of the second formation are the same, it is worse. It foretells a number of lesions arising from deficiency of mineral salts in the tissues. There is only one exception, and it is an important one. When the serrated incisors are seen in strong children, in whom the fontanelles have closed early, it is a sign of a robust constitution. Instead of a number of small and sharp serrations, there are a few large, blunt ones.

9. To regard the eruption of the teeth as the sole factor in the general process known as the first dentition, is to perpetuate a sort of medical synecdoche. Children get their first teeth because they are at the same time getting a second stomach and second intestines.

10. The body of a child possesses such a degree of "acoustic transparency" that, in cases of necessity or convenience, auscultation may be practiced with the hand, converting it into a telephone, which will reveal as much to the physician as even his ear could do.

11. In practice it is well to distinguish with precision a case in which disease is due to lumbricoids from one in which lumbricoids are due to disease.

For in the former case anthelmintics are of service, but in the latter they do harm.

12. Since, until a child is able to talk clearly, his relations with the physician are purely objective, it is very necessary that we should study as carefully as do the veterinarians the exact correspondence between the lesions and the expression of the patients.

13. If you wish to cure rapidly and well joint-diseases in infants, you must treat them as you would a conflagration—douches, douches and more douches, until you have succeeded in extinguishing them.

14. The entire system of the moral relations between children and adults should be changed. To speak to them incorrectly merely because they cannot pronounce well; to excite their fears and arouse their weird imagination simply because they are easily frightened and impressionable; to stimulate their vanity because they are naturally inclined to be vain; these and other similar actions are not only wrong, but absurd.

15. There is, finally, danger to the woman of contracting a vice as yet unregistered in the annals of concupiscence—mastomania, or the sensuality of nursing. When this physiological act degenerates into a vice, nursing becomes so frequent as to be nearly continuous, and the result is ruin to both mother and child. Finally, the physician must here, as always, be at once wise, discreet, of good judgment, and firm.—*N. Y. Med. Record.*

